# Foxi3 transcription factor activity is mediated by a C-terminal transactivation domain and regulated by the Protein Phosphatase 2A (PP2A) complex

**DOI:** 10.1038/s41598-018-35390-8

**Published:** 2018-11-22

**Authors:** Sunita Singh, Rahul K. Jangid, Alyssa Crowder, Andrew K. Groves

**Affiliations:** 1 Department of Neuroscience, Baylor College of Medicine, 1 Baylor Plaza, Houston, TX 77030 USA; 2 Department of Molecular and Human Genetics, Baylor College of Medicine, 1 Baylor Plaza, Houston, TX 77030 USA; 3 Program in Developmental Biology, Baylor College of Medicine, 1 Baylor Plaza, Houston, TX 77030 USA

## Abstract

The Forkhead box (FOX) family consists of at least 19 subgroups of transcription factors which are characterized by the presence of an evolutionary conserved ‘forkhead’ or ‘winged-helix’ DNA-binding domain. Despite having a conserved core DNA binding domain, FOX proteins display remarkable functional diversity and are involved in many developmental and cell specific processes. In the present study, we focus on a poorly characterized member of the Forkhead family, Foxi3, which plays a critical role in the development of the inner ear and jaw. We show that Foxi3 contains at least two important functional domains, a nuclear localization sequence (NLS) and a C-terminal transactivation domain (TAD), and that it directly binds its targets in a sequence specific manner. We also show that the transcriptional activity of Foxi3 is regulated by phosphorylation, and that the activity of Foxi3 can be attenuated by its physical interaction with the protein phosphatase 2A (PP2A) complex.

## Introduction

Forkhead (FOX) family transcription factors are a diverse family of regulatory proteins that play important roles in various biological processes including embryonic development, differentiation, proliferation, survival, senescence, apoptosis, migration, longevity, invasion and tumorigenesis^1–3^. FOX genes encode helix–turn–helix proteins and are characterized by the presence of an evolutionary conserved ‘Forkhead’ (FKH) or ‘winged-helix’ domain that acts as the DNA-binding domain for these factors^4,5^. The first FOX gene was originally discovered in Drosophila as the fkh gene whose mutants displayed a fork-headed phenotype^6^. Since then, many different FOX proteins have been discovered across different species^7,8^ that have been further categorized into 19 subfamilies (FOXA to FOXS) on the basis of sequence homology^9^. Despite having homology between their DNA-binding domains and their DNA-recognition motifs, Fox proteins have evolved distinct roles and regulate distinct process during development and differentiation^8,10,11^.

In mice, the FOXI family consists of 3 members - FOXI1, 2 and 3. Although they possess a forkhead DNA binding domain that defines this large gene family, *Foxi* gene family members are unusual in that the region coding for the forkhead domain is split by an intron^12^. Foxi proteins play a role in the development and function of the inner ear and other craniofacial structures. Foxi1 mutant mice exhibit vestibular dysfunction, circling behavior, and deafness^13,14^. Foxi1 has been shown to regulate expression of the Pendrin ion transporter encoded by *Slc26a4*^15^, and pathogenic human variants in both FOXI1 and SLC26A4 cause Pendred syndrome^16^. Although mouse and chicken *Foxi2* are expressed in ectoderm adjacent to the otic placode that includes the future epibranchial ganglia^17,18^, *Foxi2* null mice develop normally with no apparent morphological phenotype and can be bred as homozygous mutants^19^ (Ohyama, Edlund and Groves, unpublished observations). Induction and differentiation of the otic placode, the anlagen of the inner ear, is regulated by FOXI proteins in various species including mouse, zebrafish and Xenopus^20^. Deletion of *Foxi3* in mice causes a failure of otic placode induction and hence a complete absence of inner ear structures. In addition, the middle and outer ears and lower jaw are also reduced or absent in *Foxi3* null mice due to defects in development of the pharyngeal arches^19,21^.

*F*oxi3 has been shown to regulate several target genes during otic placode and ear development^19,21^. However, little is known about the molecular mechanisms of gene regulation by FOXI3 and its related FOXI family members, nor of its functional and regulatory domains and how their function may be modified post-translationally. The present study aims to answer these questions. Our results show that Foxi3 directly binds the target promoter in a sequence specific manner and contains a functional nuclear localization sequence (NLS) and C-terminal transactivation domain. We further show that Foxi3 activity is modified by phosphorylation of conserved serine residues, and can be attenuated by Foxi3’s physical association with the protein phosphatase 2A (PP2A) complex.

## Results

### Foxi3 regulates the *AE4* promoter

**Figure 1:**
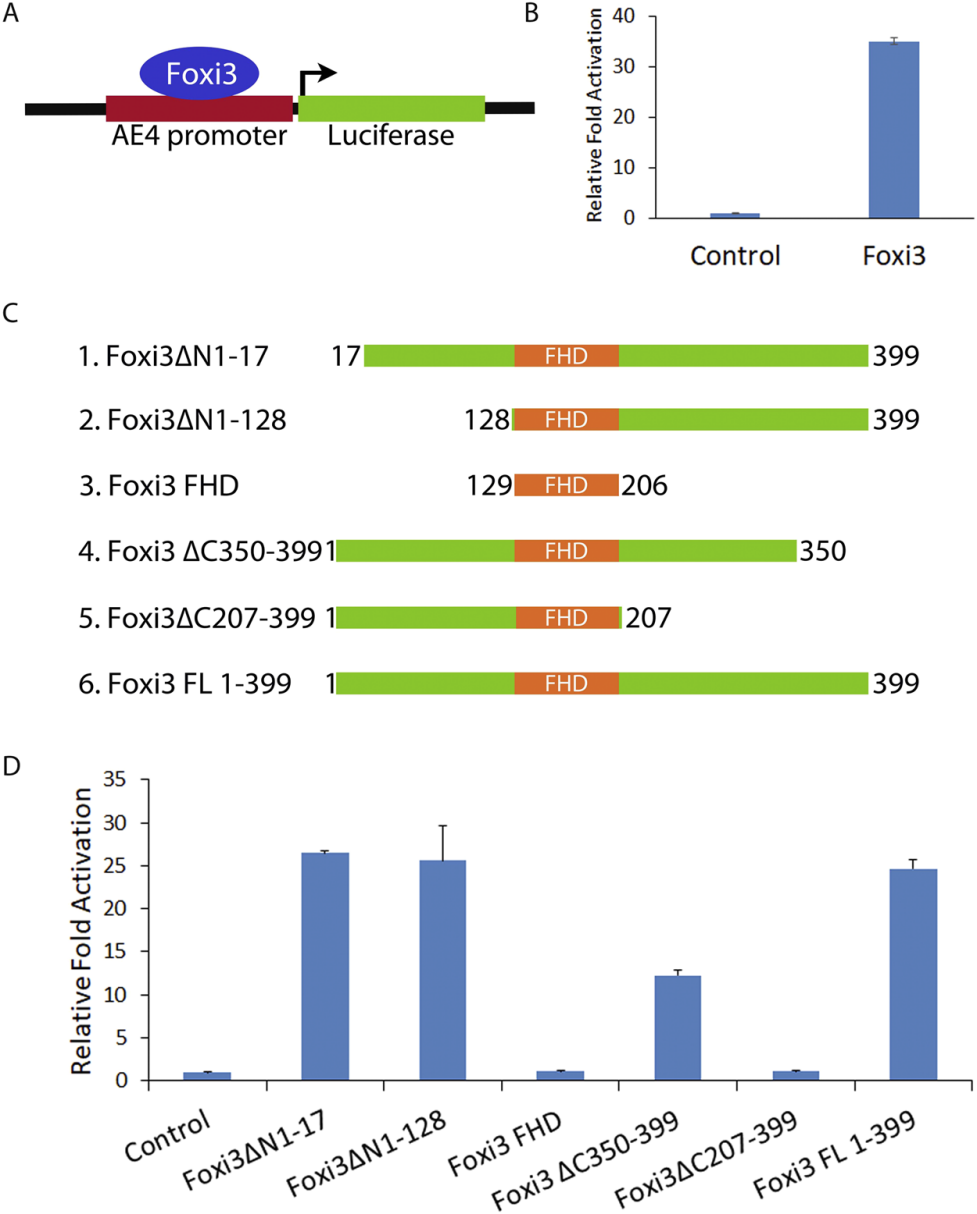
Identification of functional domains in Foxi3 by deletion analysis. (**A**) Diagram showing a reporter construct in which the Foxi1-responsive *AE4* promoter (22) is cloned upstream of a luciferase reporter gene. (**B**) Foxi3 activates transcription from the *AE4* promoter as shown by a > 30 fold activation of the luciferase reporter. (**C**) Schematic representation of various N-terminal and C-terminal truncations of Foxi3 which were cloned in 3XFLAG vector. (**D**) *AE4* promoter activity was measured after co-transfection of the *AE4* luciferase reporter with various N-terminal and C-terminal truncations of Foxi3. *AE4* promoter-linked Luciferase activity is shown as relative fold activation compared with control. Each experiment was performed in triplicate and was repeated at least three times. Error bars represent standard deviations calculated from the biological triplicates. Δ: deletion, FL: full-length, FHD: Forkhead domain.

We first sought to determine if Foxi3 can act as a transcriptional activator and which Foxi3 domains are necessary for its function. To test this, we used an *AE4* promoter that has been previously shown to be regulated by Foxi1 during the differentiation of kidney intercalated cells^22^. As Foxi3 shares significant sequence similarity to Foxi1, we asked if Foxi3 could also activate the *AE4* promoter. We cloned the *AE4* promoter into the pGL3basic vector (Fig. 1A) and performed luciferase assays in HEK-293T cells. Co-transfection of the Foxi3 with the *AE4* luciferase reporter resulted in >30 fold activation compared with an empty vector control (Fig. 1B).

To identify functional domains in Foxi3, we created a number of N-terminal and C-terminal truncations (Fig. 1C) and cloned them into a 3XFLAG vector. These Foxi3 mutants were co-transfected with the *AE4* luciferase reporter in HEK-293T cells. The Forkhead domain of Foxi3 alone did not activate the luciferase reporter, indicating that the Forkhead domain by itself is not sufficient for transcriptional activation. Removal of amino acids 1–128 from the N-terminus had no significant effect on Foxi3 activity. However, C-terminal deletion of amino acids 207–399 from C-terminus completely abolished Foxi3’s ability to activate the luciferase reporter, indicating that it contains at least one domain necessary for activation. Removal of amino acids 350–399 reduced Foxi3’s ability to activate the luciferase reporter by more than half, indicating that this domain is also necessary for Foxi3 activity (Fig. 1D).

### Foxi3 contains a functional nuclear localization sequence

**Figure 2:**
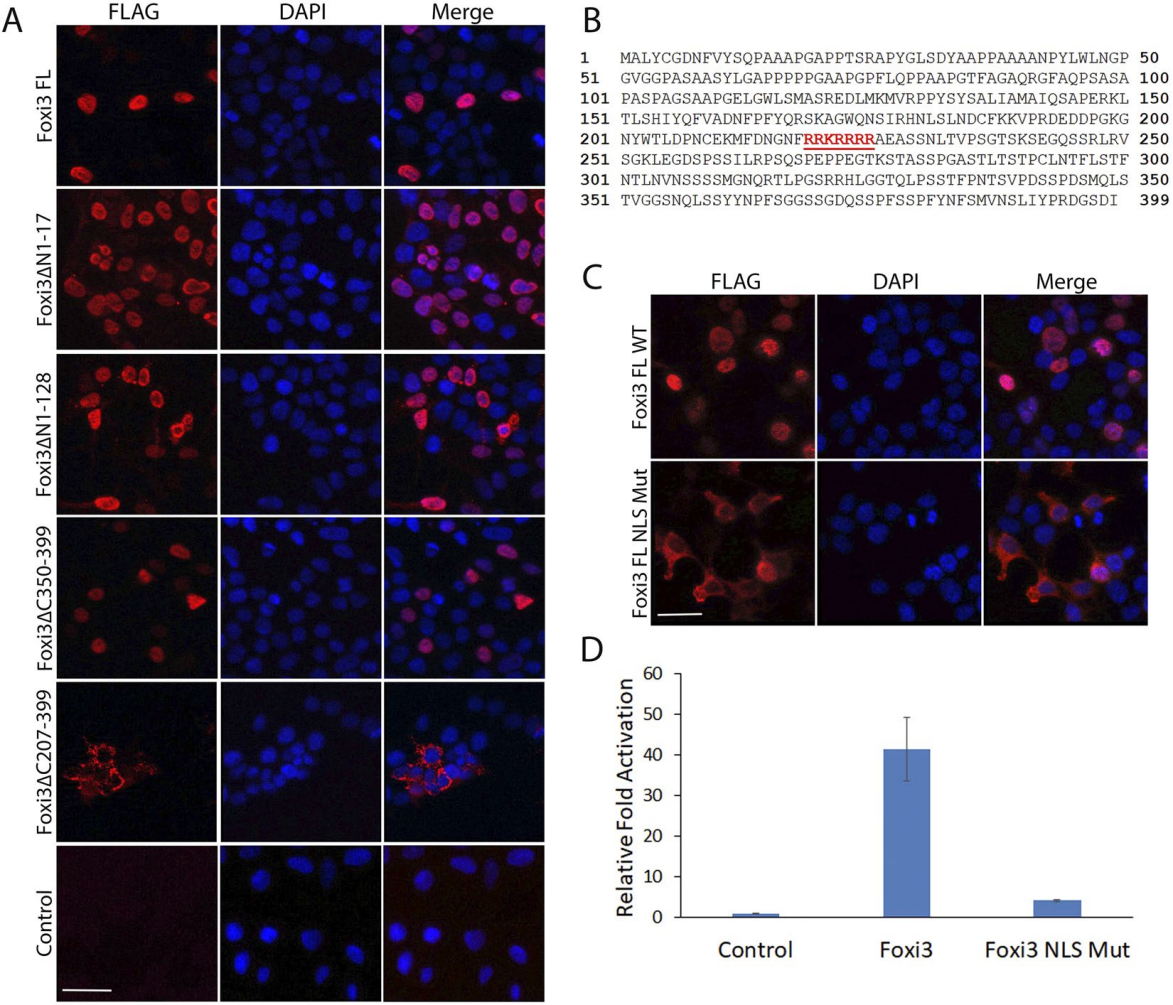
Characterization of nuclear localization sequence (NLS) in Foxi3. (**A**) Immunostaining using FLAG antibody in HEK-293T cells transfected with FLAG-tagged N-terminal and C-terminal truncations of Foxi3 described in Fig. 1. DAPI was used as nuclear stain. Deletion of the region from AA207–399 (Foxi3ΔC207–399) prevents nuclear localization. (**B**) A nuclear localization sequence (219–225aa, shown in red) was predicted for Foxi3 protein using the NucPred tool (**C**) The predicted NLS was mutated in Foxi3 (Foxi3 FL NLS Mut) and transfected in HEK-293T cells. Wild-type Foxi3 (Foxi3 FL WT) was used as control. Mutation of the predicted NLS abolishes nuclear localization. (**D**) Mutation of the predicted NLS abolishes Foxi3 activity. *AE4* promoter activity was measured after co-transfection of *AE4* luciferase reporter with wild-type Foxi3 (Foxi3) or Foxi3 with mutated NLS (Foxi3 NLS Mut). HEK-293T cells were transfected with 250 ng of *AE4* luciferase reporter along with 250 ng of either with wild-type Foxi3 (Foxi3) or Foxi3 with mutated NLS (Foxi3 NLS Mut) coding constructs as indicated. *AE4* promoter linked Luciferase activity is shown as relative fold activation compared with control. Each experiment was performed in triplicate and was repeated at least three times. Error bars represent standard deviations calculated from the biological triplicates. Δ: deletion, FL: full-length, NLS: nuclear localization sequence. Scale bars: 50 µm.

After validating the regulatory activity of various N-terminal and C-terminal truncations of Foxi3, these FLAG tagged constructs were transfected in HEK-293T cells along with full length (1–399aa) Foxi3 followed by immunostaining for FLAG to visualize the cellular localization of the Foxi3 mutants. We observed that, while most deletion constructs showed nuclear localization similar to wild-type Foxi3, C-terminal deletion of the 207–399 domain resulted in loss of nuclear localization of Foxi3 (Fig. 2A). We analyzed this Foxi3 domain for any nuclear localization sequences (NLS) using NucPred tool^23^ which predicted one NLS (219–225aa) in this domain (Fig. 2B). We mutated this predicted NLS in wild-type Foxi3 (RRKRRRR > AAAAAAA) and repeated the immunostaining along with wild-type full length Foxi3. As expected, mutation of the predicted NLS abolishes its nuclear localization (Fig. 2C) indicating that this sequence is a functional NLS. The function of this NLS is also supported by the reporter assay where Foxi3 with a mutated NLS fails to activate the *AE4* promoter (Fig. 2D).

### Foxi3 contains a C-terminal transactivation domain

**Figure 3:**
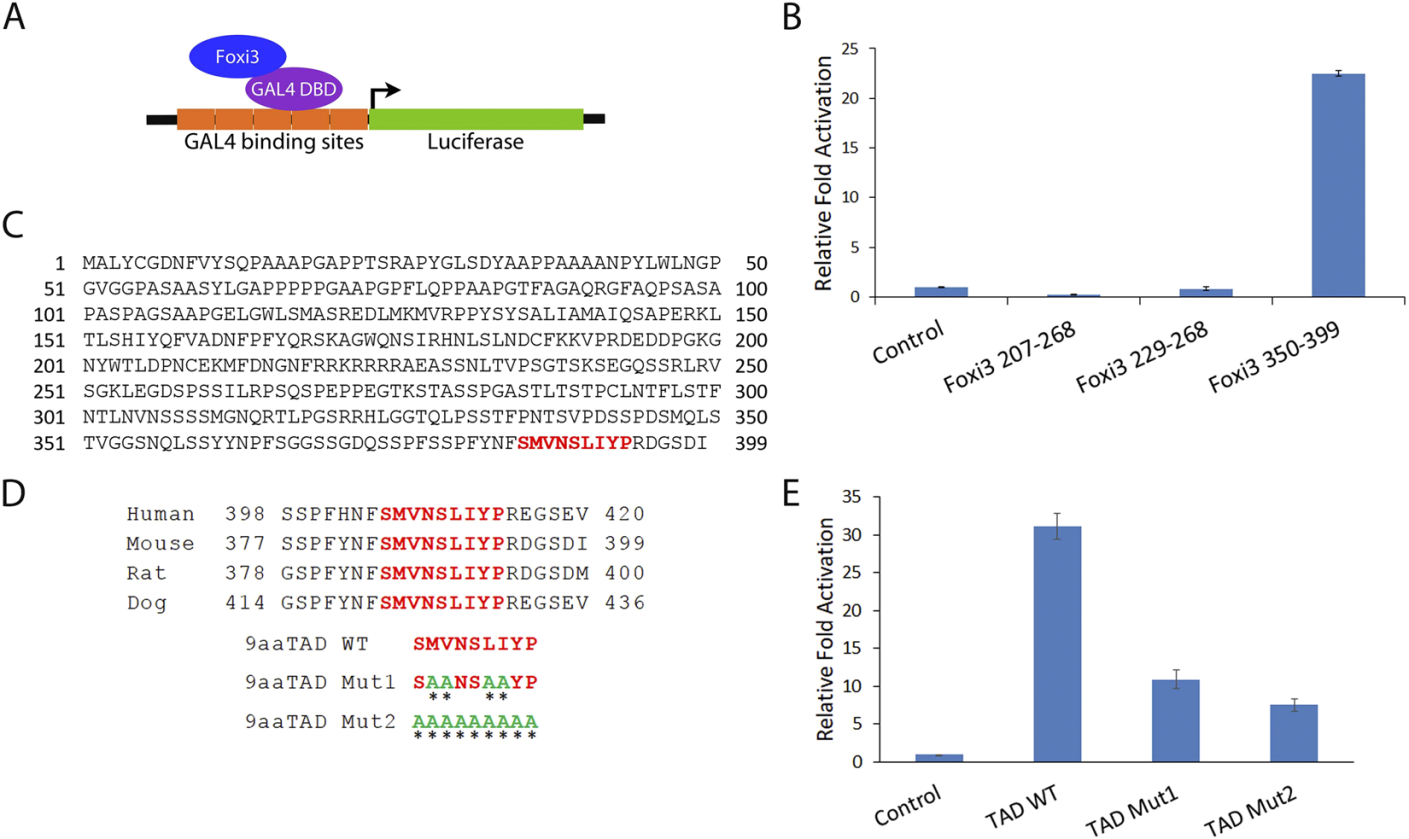
Characterization and validation of a C-terminal transactivation domain in Foxi3. (**A**) Foxi3 fragments were cloned as a fusion construct with the DNA binding domain of GAL4 (shown as GAL4 DBD). Foxi3-GAL4 DBD fusion protein binds to the GAL4 binding sites present upstream of a luciferase reporter vector. (**B**) Luciferase activity was measured after co-transfection of the luciferase reporter with only GAL4 DBD (control) or as a fusion construct of GAL4 DBD with various Foxi3 domains as indicated. Only the C-terminal fragment containing AA350–399 gave significant activation. (**C**) The Nine Amino Acids Transactivation Domain (9aaTAD) Prediction Tool predicted a 9aa transactivation domain in Foxi3 which is conserved in human, mouse, rat and dog. (**D**) The conserved 9aaTAD (shown in red) was mutated in the GAL4 DBD-Foxi3 350–399 construct. Two 9aaTAD mutations - 9aaTAD Mut1 and 2 were generated. Mutated aa are shown in green and indicated by an asterisk. (**E**) Luciferase activity was measured upon co-transfection of the luciferase reporter with only GAL4 DBD (control) or as a fusion construct of GAL4 DBD-Foxi3 350–399 containing wild-type or mutated 9aaTADdomains as indicated. Luciferase activity is shown as relative fold activation compared with control. Each experiment was performed in triplicate and was repeated at least three times. Error bars represent standard deviations calculated from the biological triplicates. DBD: DNA binding domain, TAD: transactivation domain, WT: wild type.

The C-terminal Foxi3 deletion (Foxi3 Δ350–399) results in decrease of reporter activation compared to wild-type Foxi3, indicating that this is a potential transcriptional activation domain (Fig. 1D). To test if this domain is sufficient to activate transcription, we cloned the 350–399aa Foxi3 domain as a GAL4 fusion DNA binding protein in the pBIND vector and tested its activity in a GAL4 based pG5luc reporter assay (Fig. 3A). In addition to the 350–399aa domain, we made GAL4 fusion constructs using 207–268aa and 229–268aa regions of Foxi3. We transfected these GAL4 fusion Foxi3 domains into HEK-293T cells for reporter assays along with empty vector expressing GAL4 protein alone. We observed that only the 350–399aa domain of Foxi3 was sufficient to activate reporter activity (Fig. 3B).

Next, we screened Foxi3 sequences for any predicted transactivation domains using a 9aa TAD Prediction Tool^24^. We found one strong 9aaTAD (SMVNSLIYP) in the 350–399 domain of Foxi3 (Fig. 3C). This 9aa sequence was conserved in human, mouse, rat and dog (Fig. 3D). We mutated this 9aaTAD as shown in Fig. 3D and made two mutants, TAD Mut1 and Mut2. These two 9aaTAD mutants were transfected in HEK-293T cells along with wild-type 9aaTAD clone (TAD WT) for reporter assays. Mutating the 9aaTAD results in significant reduction of reporter activation by Foxi3, indicating that this 9aaTAD acts a functional transactivation domain (Fig. 3E).

### Foxi3 binds directly to the *AE4* promoter to regulate transcription

**Figure 4:**
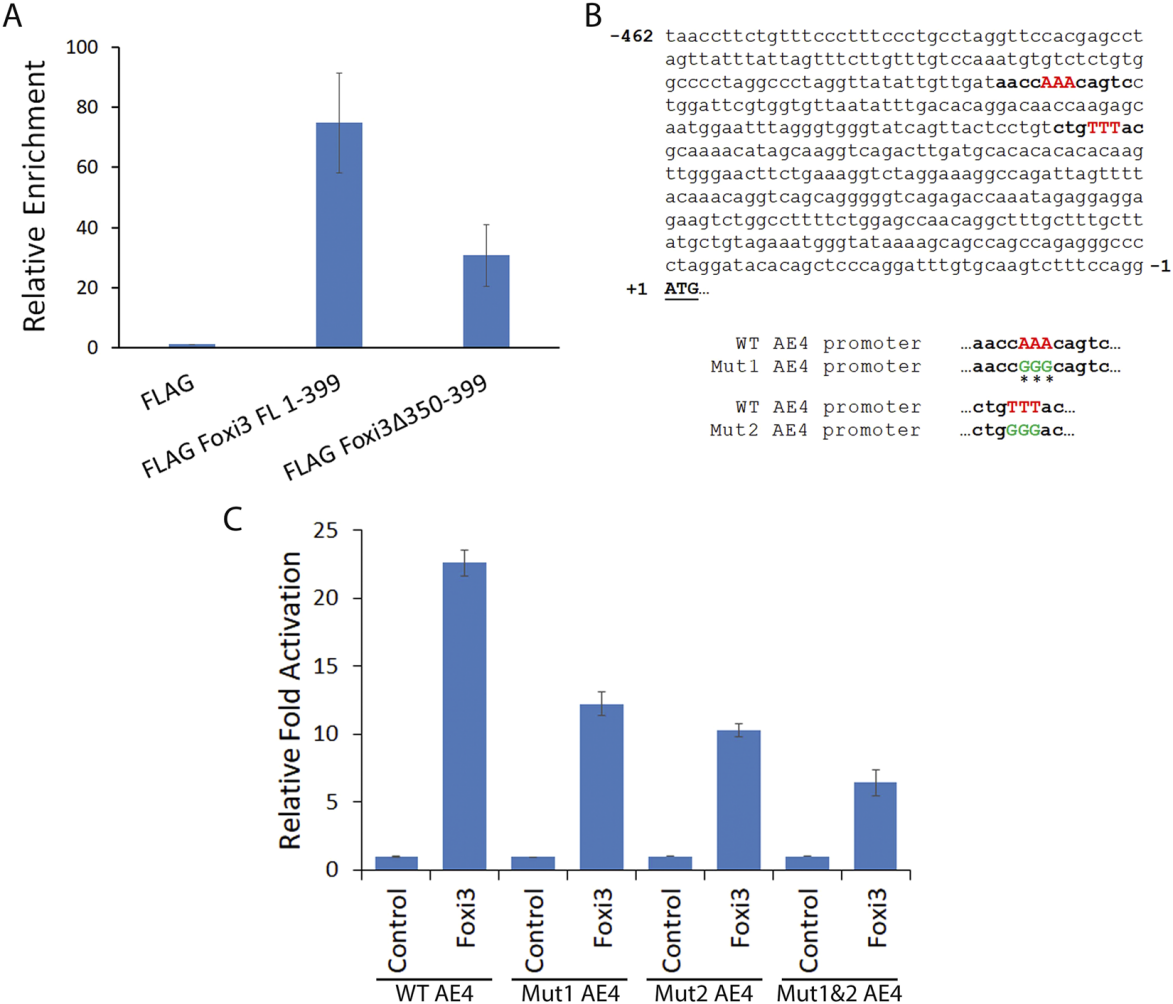
Foxi3 binds directly to the *AE4* promoter in a sequence specific manner. (**A**) Occupancy of the *AE4* promoter by Foxi3 was assayed by ChIP analysis. Chromatin was isolated from control (FLAG alone), FLAG Foxi3 FL 1–399 and FLAG Foxi3 Δ350–399 transfected HEK-293T cells and ChIP analysis was performed using Anti-Flag M2 Magnetic Beads. Relative occupancy was calculated by performing quantitative real-time PCR analysis and normalizing the CT values with input and FLAG controls. Each error bar indicates standard deviation calculated from triplicates. (**B**) The *AE4* promoter sequence contains two consensus Forkhead binding motifs ((T/C)**AAA**CA) which are indicated by bold letters. The three ‘**AAA**’ nucleotides (red) in both consensus sequences were mutated to ‘**GGG**’ (green) to create two mutant *AE4* luciferase reporter constructs labeled as Mut1 and Mut2 *AE4* respectively. Mutated nucleotides are shown in green and indicated by the asterisks. We also created a third *AE4* luciferase reporter construct where both Mut1 and Mut2 were generated in *AE4* promoter and named it as Mut1&2 *AE4*. (**C**) Luciferase activity was measured upon co-transfection of the FLAG vector or FLAG Foxi3 with wild-type (WT) *AE4*, Mut1 *AE4* and Mut2 *AE4* luciferase reporters. Mutation of one or both Forkhead binding sites significantly decreases reporter activity. HEK-293T cells were transfected with 250 ng of luciferase reporter along with 250 ng of either FLAG (control) or FLAG Foxi3 as indicated. 10 ng of Renilla Luciferase plasmid was used as an internal control in all the transfections. Luciferase activity is shown as relative fold activation compared with control. Each experiment was performed in triplicate and was repeated at least three times. Error bars represent standard deviations calculated from the biological triplicates. FL: full length, Mut: Mutation, WT: wild type.

Forkhead proteins are known to directly bind DNA to regulate transcription. To test if Foxi3 could directly bind to the *AE4* promoter, we co-transfected HEK-293T cells with an *AE4* luciferase construct and FLAG-tagged wild-type Foxi3 (FLAG Foxi3 FL 1–399) or Foxi3 with a C-terminal deletion of 350–399aa (FLAG Foxi3 Δ350–399). Since we have been unable to identify a commercial Foxi3 antibody that works well for ChIP, we performed ChIP using anti-FLAG M2 magnetic beads and analyzed the occupancy of Foxi3 on the *AE4* promoter by ChIP-qPCR. We observed that Foxi3 was significantly enriched on the *AE4* promoter. Moreover, deletion of the C-terminal 350–399aa domain results in reduction of Foxi3 occupancy on the *AE4* promoter (Fig. 4A).

While consensus binding sequence of other Foxi family members are well characterized^25,26^, very little is known about the binding motif of Foxi3. Several Forkhead proteins, including members of the Foxi family, recognize a similar conserved binding motif with the consensus (T/C)**AAA**CA^27,28^. We found two such consensus sequences in the *AE4* promoter (Fig. 4B). To test if these sequences indeed contributed to the Foxi3 mediated *AE4* gene regulation, we mutated the AAA motif in each site (shown in the red and bold in the wild type, Fig. 4B) to GGG (shown in the green and bold in the mutant, Fig. 4B). We made two independent *AE4* reporter clones for these two sites, named as mut1 and mut2. We also made a third *AE4* reporter clone which contained both the mutated consensus binding sites (mut1&2). We repeated our reporter assays with the wild-type *AE4* reporter along with these 3 mutated *AE4* reporters. Our results showed that mutating either of the consensus binding sites in the *AE4* reporter resulted in the reduced activation by Foxi3 as compared to the wild-type *AE4* reporter, and this activity was further lowered when both the sites were mutated (Fig. 4C).

### Foxi3 activity is regulated by phosphorylation

**Figure 5:**
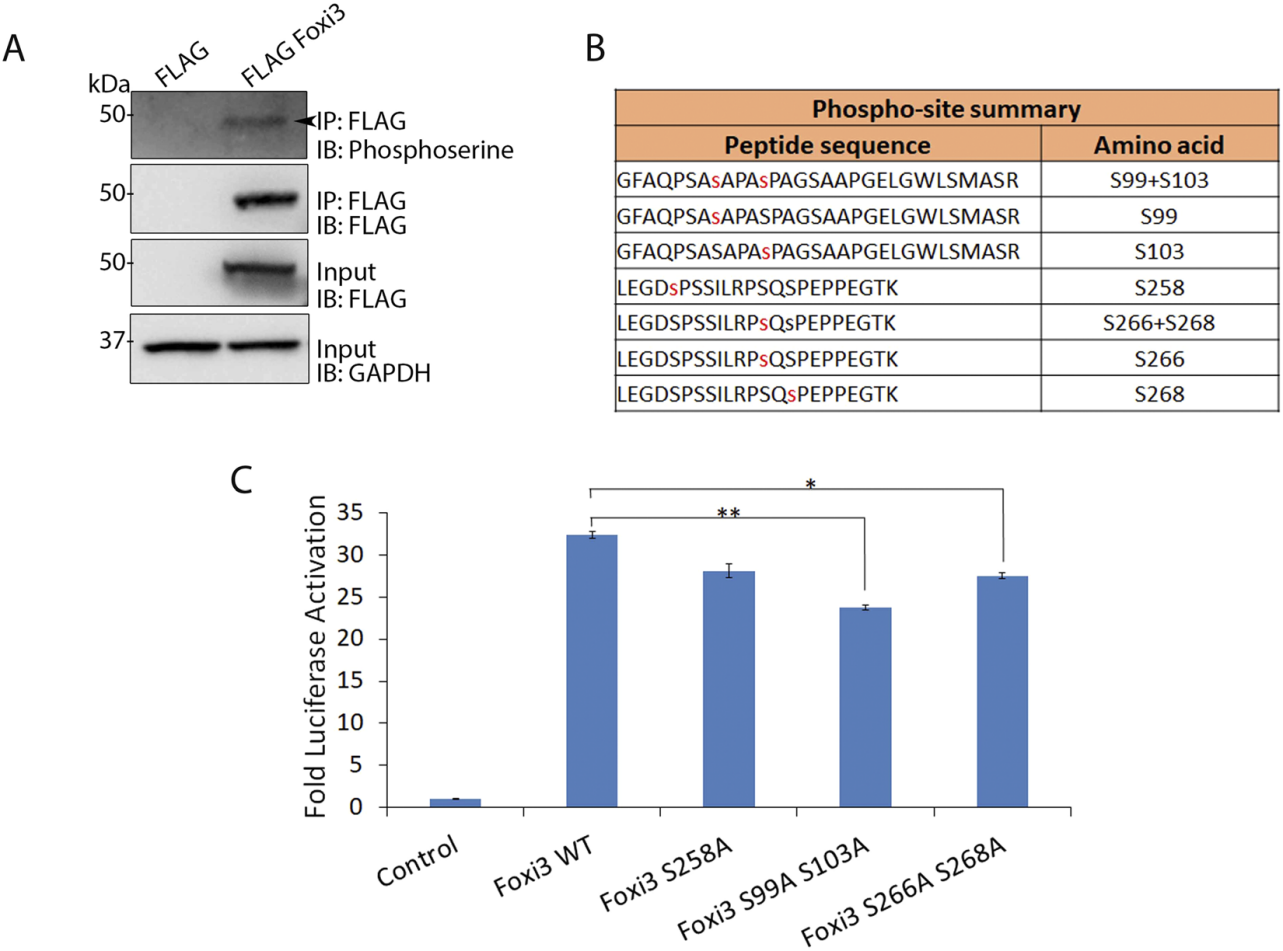
Foxi3 activity is regulated by phosphorylation. (**A**) Co-IP showing that Foxi3 is phosphorylated in HEK-293T cells. FLAG-Foxi3 was transfected in HEK-293T cells along with FLAG vector control. Anti-Flag M2 magnetic beads were used to perform immunoprecipitation followed by immunoblotting with anti-phosphoserine. Inputs are shown to validate the expression of FLAG Foxi3, and GAPDH is used to show equal amounts of lysate were used for each immunoprecipitation. Full-length blots are presented in Supplementary Fig. 2. (**B**) Diagram of the various phosphorylated serine residues identified in Foxi3 by mass spectrometry. (**C**) Mutation of serine at 99 and 103 amino acid position to alanine results in reduced Foxi3 activity. *AE4* promoter activity was measured after co-transfection of *AE4* luciferase reporter with wild-type Foxi3 (Foxi3) or Foxi3 with mutated serine residues. HEK-293T cells were transfected with 250 ng of *AE4* luciferase reporter along with 250 ng of either with wild-type Foxi3 (Foxi3) or Foxi3 serine mutant coding constructs as indicated. *AE4* promoter linked luciferase activity is shown as relative fold activation compared with control. Each experiment was performed in triplicate and was repeated at least three times. Error bars represent standard deviations calculated from the biological triplicates. Statistical significance was determined by Student’s t test. A value of P < 0.05 was considered as statistically significant (*P < 0.05; **P < 0.005). IB: Immunoblotting; IP: Immunoprecipitation; kDa: kiloDalton.

The activity of Forkhead proteins is known to be regulated by a variety of post-translational modifications (PTMs) including phosphorylation, acetylation and ubiquitination^1,2^. Phosphorylation is an important PTM which regulates the activity of different Forkhead proteins including FOXO and FOXM1^29–31^. To study if Foxi3 undergoes phosphorylation, we transfected HEK-293T cells with FLAG Foxi3 FL 1–399 and performed FLAG immunoprecipitation. A western blot of the pulldown sample with anti-phosphoserine antibodies showed that Foxi3 is phosphorylated in HEK-293T cells (Fig. 5A).

Next, we performed mass spectrometry to identify the specific phosphorylated residues in Foxi3. We identified 5 phosphorylated serine residues at 99, 103, 258, 266 and 268 amino acid positions in the Foxi3 protein (Fig. 5B). Among these, serine at 103, 258, 266 and 268 positions remain conserved between mouse and human while serine 99 is present only in mouse. We generated phospho-dead mutants for each of these 5 serine residues where serine was mutated to alanine to test the contribution of each serine residue to Foxi3 function. We transfected these Foxi3 mutants in HEK-293T cells along with the *AE4* luciferase reporter and compared their activity with wild-type Foxi3. Our results showed that mutating the serine residues at amino acids 99 and 103 resulted in significant loss of Foxi3 activity compared to wild-type Foxi3, indicating that phosphorylation plays an important role in regulating Foxi3 activity (Fig. 5C).

### Foxi3 interacts with Protein Phosphatase 2A (PP2A) subunits

**Figure 6:**
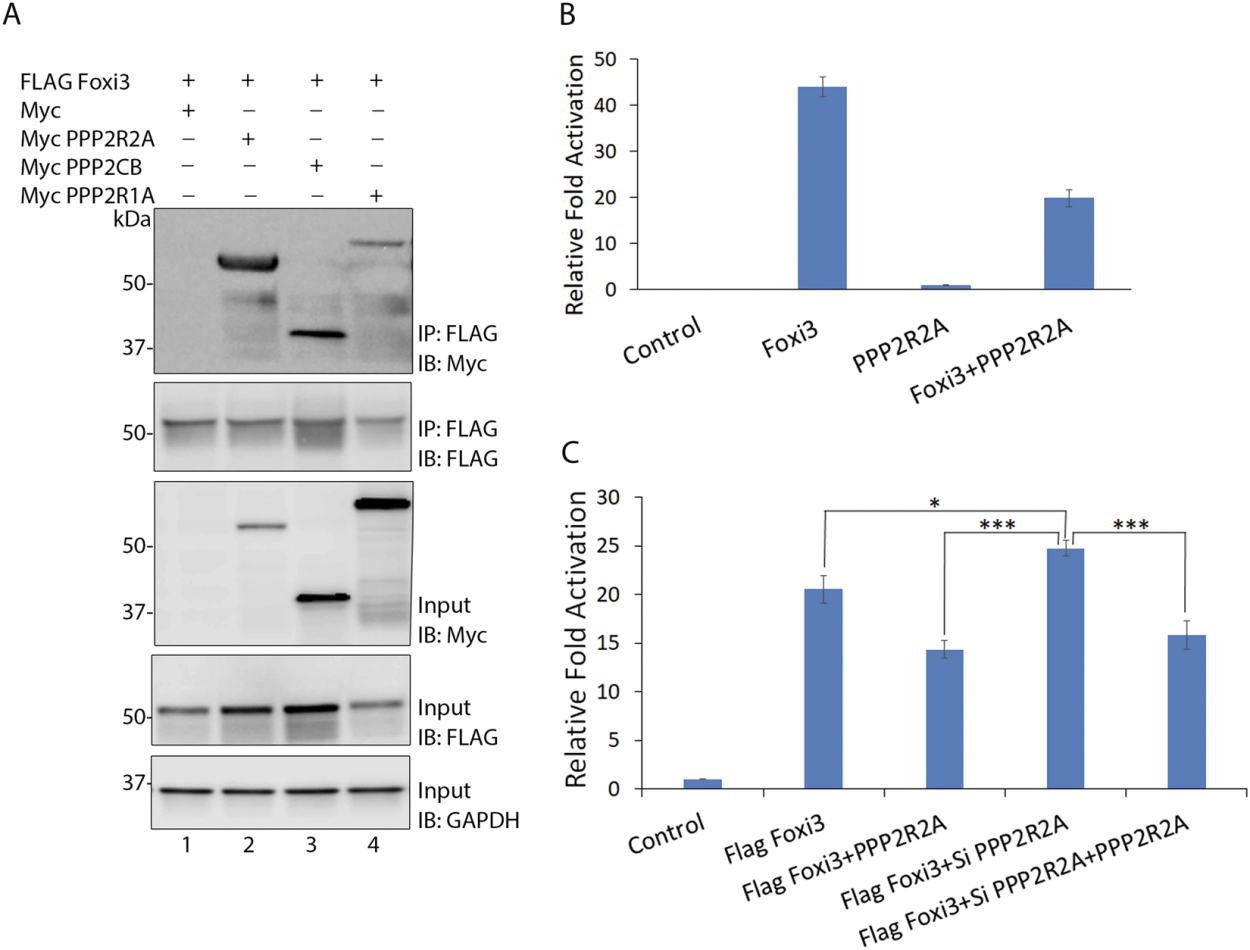
Foxi3 is negatively regulated by its interaction with the PP2A complex. (**A**) Co-IP showing the interaction between Foxi3 and various PP2A subunits. Myc-tagged PP2A subunits, PPP2R2A, PPP2CB and PPP2R1A were transfected along with FLAG Foxi3. Anti-Flag M2 magnetic beads were used to perform immunoprecipitations and MYC was used to detect the PP2A subunits. Inputs are shown to validate the expression of indicated constructs and GAPDH is used to show equal amounts of lysate were used for each immunoprecipitation. Full-length blots are presented in Supplementary Fig. 2. (**B**) Reporter assay showing that co-expression of PPP2R2A reduces the Foxi3-mediated activation of the *AE4* luciferase reporter. PPP2R2A itself does not have any effect on the reporter activity. (**C**) Expression of exogenous PPP2R2A attenuates Foxi3 reporter activation. In contrast, Foxi3-mediated reporter activation is increased by knockdown of endogenous PPP2R2A in HEK-293T cells. The effect of siRNA knockdown of PPP2R2A on Foxi3 is reversed by overexpression of a PPP2R2A cDNA. Each experiment was performed in triplicate and was repeated at least three times. P-values are calculated Results are expressed as the mean ± SD calculated from triplicates. Statistical significance was determined by Student’s t test. A value of P < 0.05 was considered as statistically significant (*P < 0.05; ***P < 0.0005). IB: Immunoblotting; IP: Immunoprecipitation; kDa: kiloDalton.

Forkhead proteins are known to regulate different biological and signaling pathways by interacting with other transcription and signaling factors^1,3,32^. To explore the interacting partners of Foxi3, we transfected HEK-293T cells with FLAG Foxi3 FL 1–399, with a blank 3XFLAG vector as a control. Whole cell lysates were prepared from the transfected cells 48 h post-transfection, and Foxi3 interacting proteins were isolated by immunoprecipitation with FLAG beads and analyzed by mass spectrometry (MS). We identified a number of transcription factors, co-factors and other nuclear proteins by MS analysis (Supplementary Table 1). One class of interacting proteins that were significantly enriched in our MS analysis were components of protein phosphatase 2A (PP2A; Table 1). PP2A is a ubiquitously expressed serine/threonine phosphatase protein complex that can exist as a dimeric core enzyme or a trimeric holoenzyme. The PP2A core enzyme is composed of a catalytic C subunit (PPP2CA or PPP2CB) and a scaffold A subunit (PPP2R1A or PPP2R1B). The dimeric core enzyme interacts with a variable regulatory B subunit (B, B’, B” or B”’) to form the holoenzyme PP2A. All these subunits are encoded by different genes, which provides a great structural diversity to the PP2A complex (Table 1)^33–35^. We identified at least one isoform of each of the 3 subunits in our MS data (Tables 1 and 2) indicating that Foxi3 might be interacting with the components of PP2A holoenzyme complex.To validate the MS data, we cloned 3 PP2A subunit isoforms, which were identified by MS and were the most abundant isoform for the respective subunit into a pCMV-Myc vector. These PP2A subunits were co-transfected with FLAG Foxi3 FL 1–399 and co-immunoprecipated using FLAG beads. Foxi3 was able to interact with all the 3 PP2A subunits (Fig. 6A).

**Table 1:** Different PP2A subunits and their isoforms along with their common name.

Subunit	Class	Isoform	Gene	Common name	Identified in MS
Scaffold	A	α	*PPP2R1A*	PR65α, PP2A-α	**Yes**
		β	*PPP2R1B*	PR65β, PP2A-β	NA
Catalytic	C	α	*PPP2CA*	PP2ACα	**Yes**
		β	*PPP2CB*	PP2Aβ	**Yes**
Regulatory	B	α	*PPP2R2A*	PR55α, PP2ABα	**Yes**
		β	*PPP2R2B*	PR55β, PP2ABβ	**NA**
		γ	*PPP2R2C*	PR55γ, PP2ABγ	**NA**
		δ	*PPP2R2D*	PR55δ, PP2ABδ	**Yes**
	B′	α	*PPP2R5A*	PR56/61α	NA
		β	*PPP2R5B*	PR56/61β	NA
		γ	*PPP2R5C*	PR56/61γ	NA
		δ	*PPP2R5D*	PR56/61δ	NA
		ε	*PPP2R5E*	PR56/61ε	NA
	B″	α	*PPP2R3A*	PR130	NA
		α	*PPP2R3A*	PR72	NA
		β	*PPP2R3B*	PR70, PR48	NA
		γ	*PPP2R3C*	G5PR	NA
		δ	*PPP2R3D*	PR59	NA
	B′′′		*STRN*	Striatin, PR110	NA
			*STRN3*	SG2NA	NA
			*PPP2R4*	PTPA, PR53	NA

The genes, which code for these isoforms in human, are also shown. Some of the isoforms that were identified in our MS data are indicated.

**Table 2:** Types of total PP2A subunits identified in the MS data and the corresponding number of peptides and percentage coverage of each subunit is shown.

Subunit	Class	Isoform	PP2A Subunit	Identified in MS	Number of peptides idetified	Protein coverage
Scaffold	A	α	PPP2R1A	**Yes**	**16**	**29.2**
Catalytic	C	α	PPP2CA	**Yes**	**3**	**50.81**
		β	PPP2CB	**Yes**	**2**	**49.19**
Regulatory	B	α	PPP2R2A	**Yes**	**21**	**68.23**
		δ	PPP2R2D	**Yes**	**5**	**23.62**

Our MS data indicated that Foxi3 interacts very strongly with PPP2R2A, a regulatory subunit isoform of the PP2A complex, compared to other regulatory B isoforms and the other catalytic and scaffold subunits. This affinity was also validated by our co-IP data (Fig. 6A). To understand how this interaction affects Foxi3 activity, we co-transfected *Foxi3* and *PPP2R2A* along with the *AE4* luciferase reporter in HEK-293T cells. Our results showed that Foxi3-mediated reporter activation is reduced when *PPP2R2A* was co-expressed with *Foxi3* (Fig. 6B). To confirm that Foxi3 association with PPP2R2A reduces its activity, we knocked down *PPP2R2A* with siRNA (Supplementary Fig. 1) and performed reporter assays by co-transfecting *Foxi3* and si PP2R2A in the presence and absence of PPP2R2A. We observed that knockdown of endogenous PPP2R2A in HEK-293T cells increased the activity of Foxi3, but co-expression of *PPP2R2A* decreased Foxi3 activity (Fig. 6C). The positive effect of PPP2R2A knockdown was reversed by titrating out the PPP2R2A siRNA with exogenous PPP2R2A cDNA.

## Discussion

Approximately fifty different Fox proteins have been identified in human and mouse, divided into 19 families^36^. Fox proteins as a group have very little sequence similarity outside the forkhead DNA binding domain, although individual families share significant sequence homology outside this region^2,8^. These conserved regions can include both transcriptional activation and repression domains^2^. Despite the importance of the *Foxi* gene family in inner ear and craniofacial development, very little is known about their transcriptional targets, whether Foxi proteins normally act as transcriptional activators or repressors, and which domains outside the forkhead DNA binding domain confer such activation or repression functions. In this study, we have identified a C-terminal region of mouse Foxi3 that contains a transcriptional activation domain, and a functional nuclear localization signal downstream from the Forkhead DNA binding domain. In addition, we present evidence that Foxi3 function can be augmented by phosphorylation. Our demonstration that Foxi3 associates with components of the PP2A complex suggests that PP2A may directly dephosphorylate Foxi3, although a definitive demonstration of this would necessitate the use of *in vitro* dephosphorylation assays.

In the present study, we identified two important functional domains in Foxi3 including a nuclear localization sequence (NLS) and a transactivation domain (TAD). Mutation of the nuclear localization sequence results in complete loss of Foxi3 activity and nuclear localization. The NLS sequence identified in Foxi3 is present immediately downstream of the Forkhead domain and is conserved in other members of FOXI family and many other Forkhead families, indicating that it is likely to act as a canonical NLS in those Forkhead protein families as well^8^. The second functional domain we identified in Foxi3 is transactivation domain (TAD) present between the 350–399aa C-terminal residues which was shown to be sufficient to induce transcription. We also identified two much shorter “9aaTADs” in this region which result in significant loss of Foxi3 activity when mutated individually or together. NLSs and TAD sequences are shown to be present in other Forkhead proteins as well^2,3^ however, this is the first report of any TAD and NLS present in Foxi3.

Forkhead protein activity is modulated by a variety of post-translational modifications in response to different signals. Some of the important modifications include phosphorylation, acetylation and ubiquitination^1,2^. Among all Forkhead proteins, modifications of the FOXO family have been studied most extensively^37–39^. FOXO proteins are regulated extensively by phosphorylation, which dictates their nuclear export, stability and transcriptional activity. Phosphorylation of FOXO3A induces its nuclear export through interaction with 14–3–3 proteins and exportins leading to its ubiquitylation and degradation^29,40^. In contrast to FOXO proteins, phosphorylation of FOXM1 induces its nuclear import and activation^31,41^. Acetylation of FOXO proteins has been shown to decrease the DNA binding ability of these proteins, while deacetylation leads to increase in transcriptional activity^30,42^. Nothing is known about the post-translational modifications of FOXI proteins. Our results show for the first time that Foxi3 is phosphorylated, and this modification is important for its transcriptional activity. We found at least 5 serine phosphosites in the Foxi3 sequence when expressed in HEK-293T cells. Among these, serine at 103, 258, 266 and 268 positions are conserved between mouse and human both while serine 99 is present only in mouse. Mutation of serine at 99 and 103 amino acid positions results in significant loss of Foxi3 activity. Moreover since these experiments are performed in human HEK-293T cells which are a heterologous system for mouse Foxi3 function, the effect of these phosho-mutants require *in vivo* experiments to demonstrate definitively. We also show that Foxi3 interacts with a serine/threonine phosphatase protein complex known as protein phosphatase 2A (PP2A). Interaction of Foxi3 and PP2R2A, a regulatory subunit isoform of the PP2A complex, results in significant reduction in Foxi3 activity indicating that phosphorylation of Foxi3 is important for its function.

*Foxi3* is expressed in the pre-placodal region (PPR) where it induces the otic placode, and in the pharyngeal region between the branchial arches. *Foxi3* is down-regulated from both the PPR and the arches as differentiation proceeds^18,19,21^. In both PPR and pharyngeal differentiation, there is evidence for FGF, Wnt and BMP signaling pathways being involved in inducing/regulating Foxi3 mRNA expression^19,20^. It is possible that dephosphorylation and phosphorylation of Foxi3 may be calibrated to tune its activity during these differentiation events. However, further investigation is required to understand if the phosphorylation we report here plays a role in the regulating Foxi3 activity during inner ear and branchial arch differentiation.

## Materials and Methods

### Antibodies and Immunostaining

Monoclonal anti-FLAG was obtained from Sigma (Sigma-F3165) and used 1:10,000 for western blotting. c-Myc antibody was obtained from Santacruz (SC-40) and used 1:1000 for western blotting. Anti-Phosphoserine antibody was obtained from Abcam (ab9332) and used 1:500 for western blotting. Anti-GAPDH antibody was obtained from Millipore (AB2302) and used 1:20,000 for western blotting.

For immunostaining, cells were fixed in 4% paraformaldehyde in PBS for 10 min at room temperature (RT), then washed once with PBS. The cells were permeabilized in PBS containing 0.25%Triton-X100 for 5 min at RT, washed once with PBS and blocked in 10% FBS in PBS for 30–60 min at RT. FLAG antibody (monoclonal anti-FLAG M2; Sigma-F3165) was diluted 1:10,000 in PBS + 3% BSA and incubated for 1 hr at 37 °C in a humidified chamber. The cells were washed three times in PBS for 5 mins each and incubated with secondary antibody (Alexa Fluor 594; Thermo-A11020; diluted 1:10,000 in PBS + 5% BSA) for 1 h at RT in the dark. The cells were washed three times in PBS for 5 mins each, stained with 10 µM DAPI for 3–5 mins, washed in PBS and mounted in Fluormount for observation and imaging.

### Cell culture, transfections and reporter assays

HEK-293T cells were maintained in DMEM with 10% Fetal Bovine Serum (Gibco) containing 100 units/ml penicillin/streptomycin in a humidified incubator at 37°C with 5% CO_2_. For reporter assays, approximately 5 × 10^4^ cells were grown overnight in 24-well plates (Corning) and transfected the next day using Lipofectamine LTX per manufacturer’s instructions (Invitrogen). Renilla luciferase plasmid (10 ng) was used as an internal control for normalization in transfection efficiencies across the wells. Cells were harvested 48 h post-transfection for reporter assays, which were performed using the Dual-Luciferase Reporter Assay System (Promega) per manufacturer’s instructions. Results were presented as the average of triplicate experiments.

### Chromatin immunoprecipitation (ChIP) assay

ChIP was performed as described in Karmodiya *et al*.^43^ with one modification in chromatin preparation for ChIP. We used MNase (NEB) digestion followed by sonication to obtain chromatin fragments of 200–300 bp. ChIP assays were performed using Anti-Flag M2 Magnetic Beads (Sigma) following the manufacturer’s instructions. For quantification of enrichment, the efficiency of chromatin immunoprecipitation was calculated by quantitative PCR (qPCR) as follows: % (ChIP/Total input) = ${2}^{{[({\rm{C}}{\rm{t}}({\rm{x}}{\rm{ \% }}{\rm{i}}{\rm{n}}{\rm{p}}{\rm{u}}{\rm{t}})-{\rm{l}}{\rm{o}}{\rm{g}}({\rm{x}}{\rm{ \% }})/{\rm{l}}{\rm{o}}{\rm{g}}2)-{\rm{C}}{\rm{t}}({\rm{C}}{\rm{h}}{\rm{I}}{\rm{P}})]}^{2}}\times 100{\rm{ \% }}$. Relative occupancy or enrichment was calculated as a ratio of specific signal over background: Enrichment = % input (specific loci)/% input (background loci). Relative enrichment was used as a measure of protein association with the AE4 enhancer.

### DNA Constructs

The promoter region of the *AE4* gene^22^ was amplified by PCR from mouse genomic DNA and cloned into the pGL3-Basic (pGL3b) vector (Promega). pGL3b-*AE4* mutant constructs were generated by site directed mutagenesis of one or both **TTT** > **GGG** sequences in the promoter. N-terminal and C-terminal truncations of Foxi3 as well as full length (FL; 1–399) Foxi3 were amplified from pCMV-SPORT6-Foxi3^44^ and cloned in a 3XFLAG vector (Sigma). A Foxi3 FL NLS mutant construct was generated by site directed mutagenesis of the predicted nuclear localization sequence (NLS) in 3xFLAG Foxi3 FL. C-terminal Foxi3 fragments were amplified and cloned as a fusion construct with the DNA binding domain of GAL4 in pBIND vector (Promega). 9aaTAD Mut1 and 2 were generated by site directed mutagenesis of the predicted 9aaTAD in pBIND Foxi3 350–399 construct. *PPP2R2A*, *PPP2CB*, *PPP2R1B* was amplified by PCR using mouse cDNA and cloned in pCMV-Myc vector (Clontech). All cloning was performed with an In-Fusion HD Cloning kit (Clontech).

### Identification of Foxi3 protein complex using nanoHPLC-MS/MS

The FLAG beads obtained after IP from FLAG Foxi3 lysates were washed and boiled with 1X Nupage™ LDS sample buffer with 50 mM 2-mercaptoenthanol and were subjected to 4–20% Tris/Glycine SDS-PAGE (Novex Gel, Invitrogen). The gels were stained with Coomassie Brilliant blue and visualized proteins bands were excised according to molecular weight. The gel pieces were subject to in-gel digestion using trypsin (GenDepot T9600). The tryptic peptide was dried under vacuum and resuspended in 10 µl of loading solution (5% methanol containing 0.1% formic acid). Half of the resuspended peptide was analyzed on a nano-HPLC 1000 system (Thermo Scientific) coupled to a LTQ Orbitrap Elite™(Thermo Scietific) mass spectrometer as recently described^45^. Parent MS spectra were acquired in the Orbitrap with full MS range of 375–1300 m/z in the resolution of 240,000.MS/MS spectra were searched against a target-decoy Human refseq database (release June 2015, containing 73637 entries) in Proteome Discoverer 1.4 interface (Thermo Fisher) with Mascot algorithm (Mascot 2.4, Matrix Science). Variable modification of oxidation of methionine and protein n-terminal acetylation was allowed. The precursor mass tolerance was confined to 20 ppm with a fragment mass tolerance of 0.5Da and a maximum of two missed cleavages allowed. Assigned peptides were filtered with 1% false discovery rate (FDR). The iBAQ algorithm was used to calculate protein abundances using an in-house data processing algorithm^45^. The mass spectrometry data have been deposited to the ProteomeXchange Consortium (http://proteomecentral.proteomexchange.org) via the MASSIVE repository (MSV MSV000082492) with the dataset identifier PXD010206.

### Identification of post-translational modifications of Foxi3

Samples were boiled in 1X NUPAGE® LDS sample buffer (Invitrogen) and subjected to SDS-PAGE (NuPAGE 10% Bis-Tris Gel, Invitrogen), and visualized with Coomassie Brilliant blue stain. The SDS-PAGE gel containing the Foxi3 band was excised, destained and subjected to in-gel digestion with 100 ng of trypsin (GenDepot T9600). Digested peptides were resuspended in 10 µl of 0.1% formic acid and analyzed with a nanoHPLC-MS/MS system with an EASY-nLC™ 1200 coupled to Fusion Tribrid Orbitrap™ Lumos™ (Thermo Scietific) mass spectrometer as recently described^45^. Parent MS spectra were acquired in the Orbitrap with full MS range of 300–1400 m/z in the resolution of 120,000. CID fragmented MS/MS spectrum was acquired in ion-trap with rapid scan mode. Obtained MS/MS spectra were searched as described above. Variable modification of phosphorylation on serine, threonine and tyrosine, oxidation on methionine, and protein N-terminal acetylation was allowed. The peptides identified from mascot result file were validated with 5% false discover rate (FDR) and subject to manual verification to confirm serine phosphorylation.

### Immunoprecipitation

Cells were washed once with PBS and lysed for 20 min in CoIP lysis buffer (Tris-HCl 25 mM, pH 7.5, NaCl 150 mM, EDTA 1 mM, NP-40 1%, Glycerol 5%, DTT 1 mM and phosphatase inhibitors) on ice. Lysates were centrifuged for 15 min at 4 °C to collect the supernatant. Anti-Flag M2 magnetic beads (Sigma- M8823-1ML) were equilibrated with CoIP lysis buffer by washing twice. The lysate was quantified and 500 µg lysate was used for each immunoprecipitation. The volume was made up to 500 µl by adding CoIP lysis buffer containing phosphatase inhibitors, and 10 µl equilibrated anti-Flag M2 magnetic beads were added to each tube which were then incubated on an end-on rocker at 4 °C overnight. After binding, the magnetic beads were collected by placing the tube in the magnetic separator. The captured beads were washed 3 times with wash buffer (Tris-HCl 10 mM, pH 7.5, NaCl 150 mM, EDTA 0.5 mM). After the last wash, the beads were resuspended in 20 µl PBS and 5 µl of 6X SDS sample buffer was added. The resuspended beads were boiled for 5–8 minutes to elute the protein complexes. The beads were collected by placing tubes in the magnetic separator and the eluates were carefully transferred to the fresh tubes which were then loaded on a SDS-PAGE gel for further analysis.

### siRNAs

siRNAs for *PPP2R2A* were purchased from Qiagen (si02225825). siRNA was transfected into HEK-293T cells at a final concentration of 20 nM using Dharmafect1 as per manufacturer’s protocol. Cell lysates were analyzed 72 h post transfections. For reporter assays, DNAs were transfected 24 h after siRNA transfection using Lipofectamine LTX.

## Electronic supplementary material

Supplementary data
