# Supplementary material for: Foxi3 transcription factor activity is mediated by a C-terminal transactivation domain and regulated by the Protein Phosphatase 2A (PP2A) complex
Source: Sci Rep. 2018 Nov 22;8:17249. doi: 10.1038/s41598-018-35390-8 (PMC6250667; doi:10.1038/s41598-018-35390-8)
Supplement: Supplementary file 1 — Supplementary data [file 41598_2018_35390_MOESM1_ESM.docx]

Foxi3 transcription factor activity is mediated by a C-terminal transactivation domain and regulated by the Protein Phosphatase 2A (PP2A) complex

Sunita Singh^1^, Rahul K. Jangid^1^, Alyssa Crowder^1^ and Andrew K. Groves^1,2,3,^*

^1^Department of Neuroscience, ^2^Department of Molecular and Human Genetics, ^3^Program in Developmental Biology, Baylor College of Medicine, 1 Baylor Plaza, Houston, TX 77030

| **Proteins enriched in MS with FLAG Foxi3** | **Number of peptides identified** |
| --- | --- |
| PPP2R2A | 27 |
| FOXI3 | 11 |
| FOXI1 | 9 |
| FOXI2 | 9 |
| TIMM50 | 18 |
| PPP2CA | 14 |
| SLC25A1 | 5 |
| STUB1 | 11 |
| LRCH2 | 1 |
| GYS1 | 19 |
| PPP2CB | 13 |
| SLC25A22 | 6 |
| SSRP1 | 7 |
| SNRPB2 | 5 |
| PPP2R1A | 15 |
| RPL39 | 1 |
| TUBA1C | 24 |
| SEC61A1 | 6 |
| PDHB | 6 |
| WDHD1 | 2 |
| POLDIP2 | 6 |
| HSPA4L | 15 |
| HPRT1 | 1 |
| RAB35 | 2 |
| GYS2 | 2 |
| MAGED1 | 4 |
| MRPS7 | 2 |
| SLC25A10 | 10 |
| AURKA | 4 |
| MSH2 | 7 |
| TARS2 | 7 |
| RAF1 | 5 |
| STT3A | 2 |
| PRKDC | 10 |
| HSPA6 | 13 |
| HNRNPH2 | 9 |
| ACACA | 3 |
| ATP5F1 | 3 |
| FARSA | 5 |
| HSPH1 | 20 |
| UBAC2 | 3 |
| GARS | 4 |
| SFXN4 | 2 |
| XPO1 | 10 |
| HSPA1L | 14 |
| PARP1 | 38 |
| HSPA8 | 40 |
| HSPA4 | 20 |
| HSPA1A | 32 |
| HSPA1B | 32 |

**Supplementary Table 1.** Top 50 enriched proteins by FLAG Foxi3 immunoprecipitation which were identified in mass spectrometry data.

**
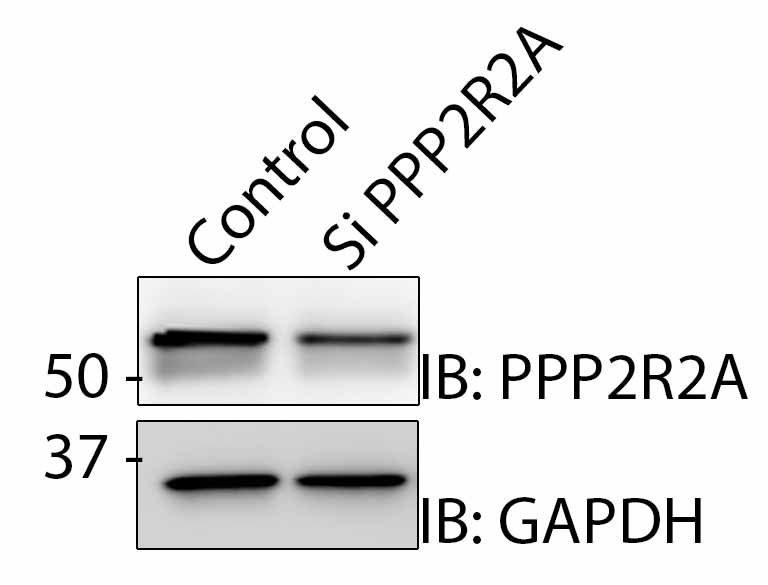
**

**Supplementary Figure 1.** Western blot showing partial PPP2R2A knockdown with an siRNA against PPP2R2A. GAPDH was used as an internal control.

**Raw data for full-length blots:**

**
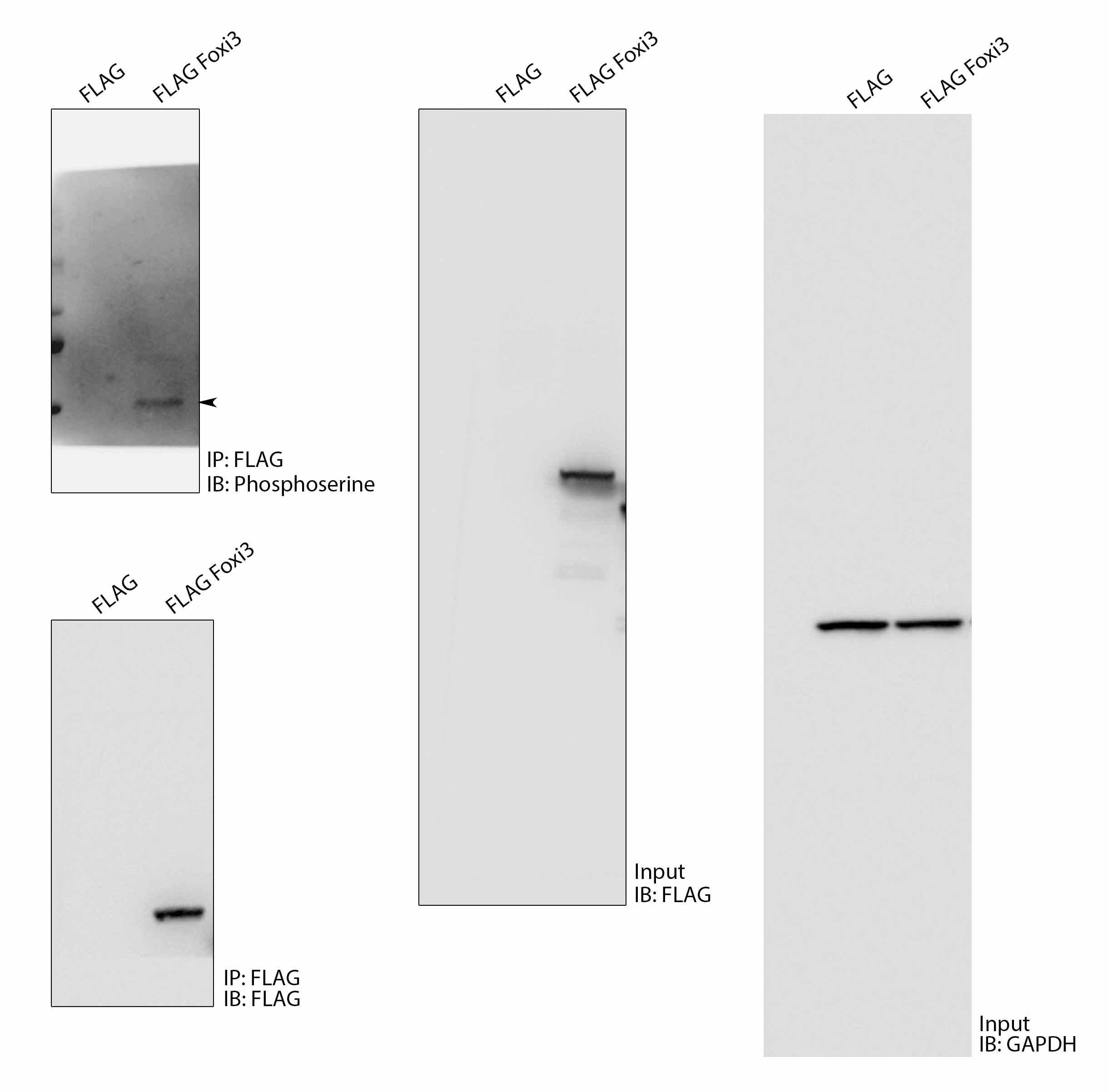
**

**Supplementary Figure 2. Full-length blots for Figure 5A**

**
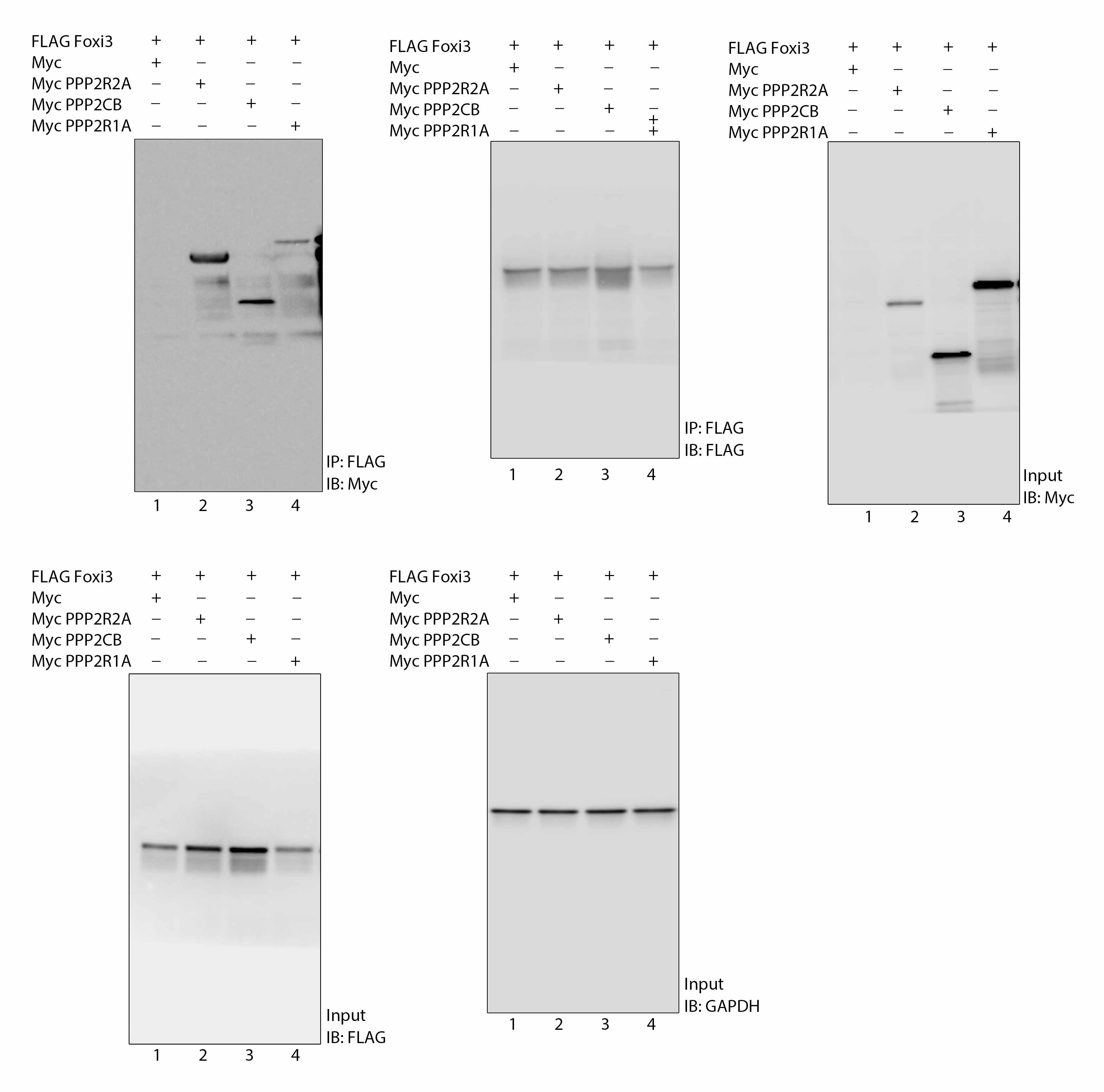
**

**Supplementary Figure 3. Full-length blots for Figure 6A**

**
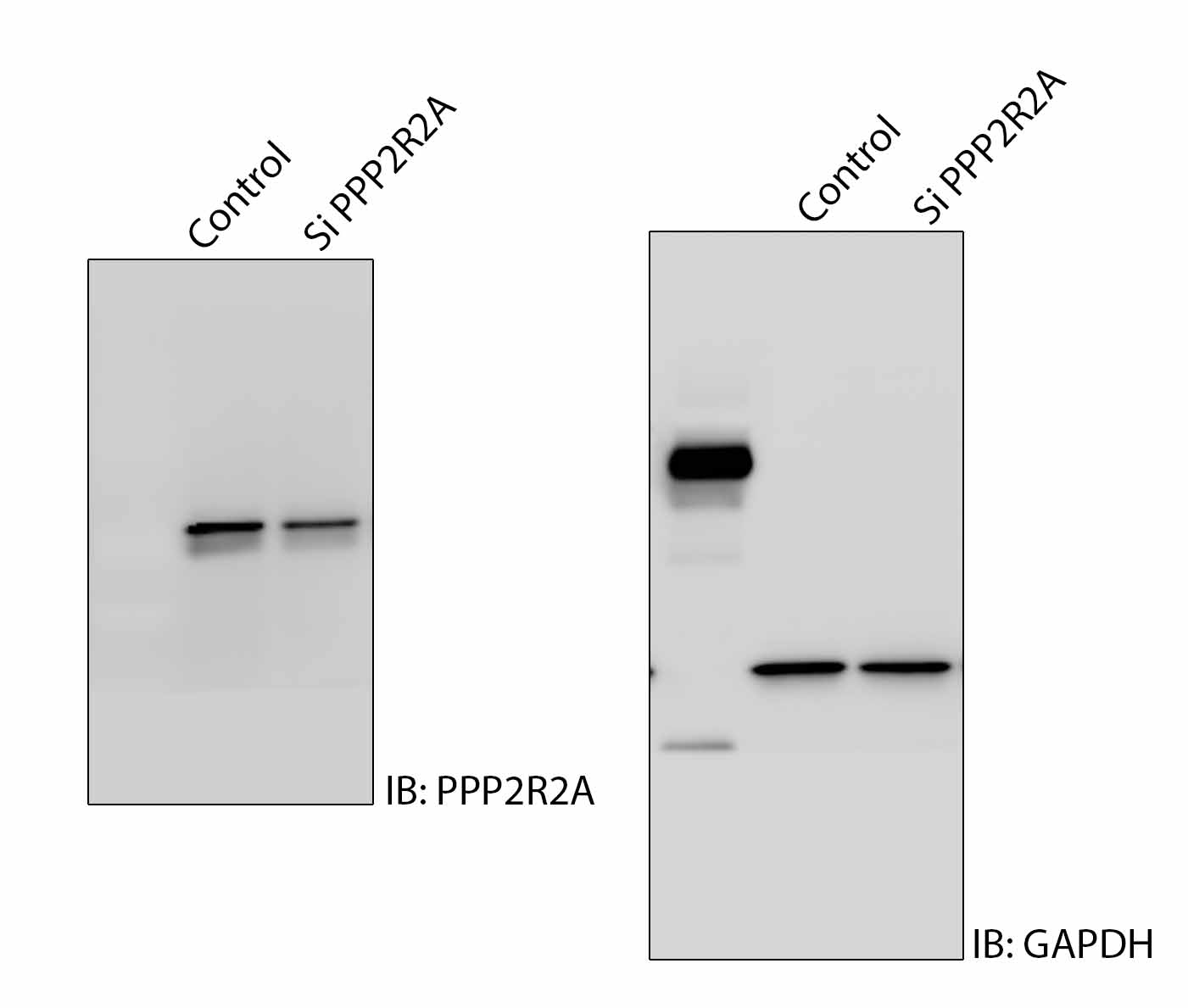
**

**Supplementary Figure 4. Full-length blots for Supplementary figure 1**
